# Mitochondrial Disease-related Mutation G167P in Cytochrome *b* of *Rhodobacter capsulatus* Cytochrome *bc*_1_ (S151P in Human) Affects the Equilibrium Distribution of [2Fe-2S] Cluster and Generation of Superoxide*

**DOI:** 10.1074/jbc.M115.661314

**Published:** 2015-08-05

**Authors:** Arkadiusz Borek, Patryk Kuleta, Robert Ekiert, Rafał Pietras, Marcin Sarewicz, Artur Osyczka

**Affiliations:** From the Department of Molecular Biophysics, Faculty of Biochemistry, Biophysics and Biotechnology, Jagiellonian University, 30-387 Kraków, Poland

**Keywords:** bioenergetics, electron transfer, enzyme kinetics, mitochondrial disease, reactive oxygen species (ROS), respiratory chain, superoxide ion, cytochrome bc1

## Abstract

Cytochrome *bc*_1_ is one of the key enzymes of many bioenergetic systems. Its operation involves a large scale movement of a head domain of iron-sulfur protein (ISP-HD), which functionally connects the catalytic quinol oxidation Q_o_ site in cytochrome *b* with cytochrome *c*_1_. The Q_o_ site under certain conditions can generate reactive oxygen species in the reaction scheme depending on the actual position of ISP-HD in respect to the Q_o_ site. Here, using a bacterial system, we show that mutation G167P in cytochrome *b* shifts the equilibrium distribution of ISP-HD toward positions remote from the Q_o_ site. This renders cytochrome *bc*_1_ non-functional *in vivo*. This effect is remediated by addition of alanine insertions (1Ala and 2Ala) in the neck region of the ISP subunit. These insertions, which on their own shift the equilibrium distribution of ISP-HD in the opposite direction (*i.e.* toward the Q_o_ site), also act in this manner in the presence of G167P. Changes in the equilibrium distribution of ISP-HD in G167P lead to an increased propensity of cytochrome *bc*_1_ to generate superoxide, which becomes evident when the concentration of quinone increases. This result corroborates the recently proposed model in which “semireverse” electron transfer back to the Q_o_ site, occurring when ISP-HD is remote from the site, favors reactive oxygen species production. G167P suggests possible molecular effects of S151P (corresponding in sequence to G167P) identified as a mitochondrial disease-related mutation in human cytochrome *b*. These effects may be valid for other human mutations that change the equilibrium distribution of ISP-HD in a manner similar to G167P.

## Teaser

**Background:** Mutation S151P was found in patients with exercise intolerance.

**Results:** Bacterial analogous substitution (G167P) influences movement of the iron-sulfur protein head domain (ISP-HD), increasing ROS production.

**Conclusion:** This correlation corroborates the recently proposed “semireverse” electron transfer mechanism of ROS production.

**Significance:** The molecular effect identified for S151P may be valid for several other human mutations that affect motion of ISP-HD.

## Introduction

Cytochrome *bc*_1_ is a key component of the electron transport chain (for a recent review, see Ref. 1). It catalyzes the reaction of reduction of cytochrome *c* by quinol. Its action is related to building a proton-motive force, which is utilized to produce ATP. As a part of the mitochondrial electron transport chain, cytochrome *bc*_1_ (complex III) plays a crucial role in oxidative phosphorylation (2).

**FIGURE 1.**
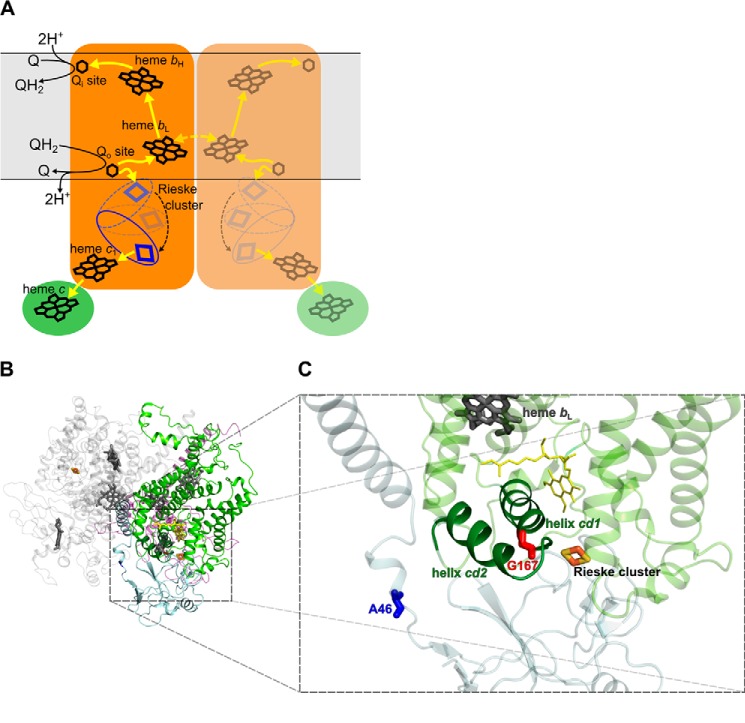
*A*, a simplified scheme of reactions occurring in dimeric cytochrome *bc*_1_. Cytochrome *bc*_1_ catalyzes reduction of cytochrome *c* (*green ellipse*) by quinol. The Q cycle reactions are highlighted for one monomer (*left*, *orange shape*). *Yellow arrows* designate the electron transfer pathway. For simplicity, only forward reactions are shown. The *yellow dotted arrow* represents intermonomer electron transfer between the *b*_L_ hemes (57, 58). A domain harboring Rieske cluster (*blue*) moves (*black dotted arrow*) between Q_o_ position and *c*_1_ position. The membrane is shown as a *light gray* area. *B*, crystal structure of dimeric cytochrome *bc*_1_ from *R. capsulatus* (Protein Data Bank code 1ZRT (59)). Subunits of one monomer are colored as follows: cytochrome *b*, *light green*; ISP, *gray*; cytochrome *c*_1_, *transparent blue*. Subunits of the second monomer are in *light gray. C*, Close up view of the part of cytochrome *b* and ISP showing structural details of the Q_o_ site. Gly-167 (*red sticks*) in cytochrome *b* (*light green*) is located in the end of helix *cd1* (*dark green*). Ala-46 (*blue sticks*) located in the neck region of ISP (*gray*) indicates the position where one (1Ala) or two (2Ala) alanine residues were inserted. *Dark gray* and *light orange sticks* indicate heme *b*_L_ and Rieske cluster, respectively. *Yellow lines* represent the stigmatellin molecule.

In the catalytic Q cycle of cytochrome *bc*_1_ (3, 4), energy connected with quinol to cytochrome *c* electron flow is utilized for proton translocation. In this case, electrons derived from the quinol oxidation are split at the Q_o_ catalytic site into two different cofactor chains: high potential c-chain (which includes Rieske cluster, heme *c*_1_, and heme *c* embedded in iron-sulfur protein (ISP),^3^ cytochrome *c*_1_, and diffusible substrate cytochrome *c*, respectively) and low potential b-chain (which includes heme *b*_L_, heme *b*_H_, and the quinone reduction Q_i_ site embedded in cytochrome *b* subunit) (Fig. 1*A*). Electrons transferred via the b-chain reduce quinone in the Q_i_ site in two sequential steps (5). Transfer of electrons via the c-chain is made possible by movement of the head domain of iron-sulfur protein (ISP-HD), an important mechanistic element of the cytochrome *bc*_1_. ISP-HD moves between a site on the cytochrome *b* interface close to the Q_o_ site (Q_o_ position) and a site on the cytochrome *c*_1_ interface (*c*_1_ position) (6–11). Unstable semiquinone is frequently discussed as an intermediate of the Q_o_ site reaction (12–16). Recent experiments have trapped semiquinone in this site in the state of a free radical (17–19) or as a semiquinone coupled to the reduced Rieske cluster (19).

However, under certain conditions, part of the energy released from oxidation of quinol can be dissipated through side reactions that result in partial or total loss of proton translocation function of cytochrome *bc*_1_. These reactions reduce the efficiency of separation of electrons in the Q_o_ site into two cofactor chains. One of the possible side reactions is an electron transfer from semiquinone generated in the Q_o_ site to oxygen to produce superoxide (15, 20, 21). This reaction causes a decrease in the yield of proton translocation and results in formation of free radicals that could cause damage to certain macromolecules including lipids and proteins.

Under some specific conditions, when the electron flow through cofactor chains is impeded, the side reactions in which superoxide is generated may be enhanced. For example, the presence of antimycin blocks the electron flow through the Q_i_ site (20–23) and favors reverse electron transfer from heme *b*_L_ to quinone with formation of semiquinone in the Q_o_ site (24, 25). The formed semiquinone can react with ISP if ISP-HD is at the Q_o_ position, or it can react with oxygen if ISP-HD is not present in the Q_o_ site. Therefore states of the Q_o_ site with ISP-HD not present at this site favor superoxide production (25, 26).

Among all subunits of complex III, only cytochrome *b* is encoded by mtDNA. Thus the probability of the occurrence of mutations in this subunit is higher compared with other subunits of the complex encoded by nDNA. In principle, such mutations may have various effects on function including adaptive effects proposed for mutations in humans: T15204C (I153T), T14798C (F18L), and G15257A (D171N) found in haplogroup C, J1, and J2, respectively (27). Nevertheless, most of the mutations identified so far in human cytochrome *b* appear to be linked with diseases such as exercise intolerance, myopathy, and cardiomyopathy (28–31). They all are somehow related to reduced efficiency of energy conversion, which could be caused by altered operation of mutated complex III. However, studying the molecular effects of these mutations in humans is often difficult, especially in the context of the occurrence of heteroplasmy and limited amount of protein that can be obtained from human cells. Those types of limitations can be overcome when using bacterial (32, 33) or yeast (30, 34) systems, which thus provide a good model to study human mitochondrial disease-related mutations at the molecular level. Conversely, the identified effects of certain human mutations can be a valuable source of general information on mechanisms of catalytic and side reactions.

This approach was undertaken in the present study. We chose mutation T15197C (in the mtDNA sequence) identified in patients with exercise intolerance (35). This mutation changes serine 151 to proline in the vicinity of the Q_o_ site and was found to result in expression of complex III with slightly reduced amounts of cytochrome *b* and cytochrome *c*_1_ (35). In a yeast model, the analogous mutation (S152P) resulted in expression of complex III with significantly reduced amounts of ISP subunit (29). We show that this mutation introduced at an analogous position to the cytochrome *b* subunit (G167P) of bacterial cytochrome *bc*_1_ influences the average position of ISP-HD with respect to other subunits in such a way that it stays more remote from the Q_o_ site than in the native enzyme. At the same time, no changes in the amounts of subunits in the catalytic core were observed. This provided new insight into the possible molecular basis of the human disease associated with the presence of T15197C. It also provided the first (to our knowledge) mutational variant of cytochrome *bc*_1_ in which a shift of ISP out of the Q_o_ site was identified spectroscopically. This allowed us to extend the array of conditions that tested changes in the levels of superoxide production by cytochrome *bc*_1_ toward a better understanding of the mechanism of this reaction.

## Experimental Procedures

### 

#### 

##### Preparation of Mutants

To construct mutations in cytochrome *bc*_1_, a genetic system originally developed by Dr. F. Daldal (University of Pennsylvania, Philadelphia, PA) (36) was used. G167P mutation (underlined) was introduced in the gene coding for cytochrome *b* (*pet*B) using the QuikChange site-directed mutagenesis system (Stratagene) and the following PCR primers: G167P_FWD (5′-C ACC GGC CTG TTT CCG GCG ATC CCG GGC ATC G-3′) and G167P_REV (5′-GCC CGG GAT CGC CGG AAA CAG GCC GGT GAT CAC G-3′). To construct single mutant G167P, pPET1 plasmid containing wild type (WT) *pet*ABC operon was used as a template DNA. To obtain G167P/1Ala and G167P/2Ala double mutants, the template DNA contained appropriate GCG insertions (resulting in 1Ala or 2Ala) in the *pet*A gene coding for ISP (10). The correct sequence of engineered constructs was verified by sequencing entire *pet*A and *pet*B genes. The BstXI-XmaI fragments of the operon containing the desired mutations and no other mutations were exchanged with WT counterpart of pMTS1 plasmid. Expression vectors were introduced into MT-RBC1 *Rhodobacter capsulatus* strain (devoid of *pet*ABC operon) using triparental crossing (36). The presence of introduced mutations was confirmed by sequencing the *pet*A and *pet*B genes on a plasmid isolated from the mutated *R. capsulatus* strains.

*R. capsulatus* bacteria were grown under semiaerobic or photoheterotrophic conditions as described previously (37). To test for the occurrence of reversion mutations, 100 μl of 2 ml of overnight liquid culture of the mutant strains were spread on mineral-peptone-yeast extract (MPYE) plates and kept in selective photosynthetic cultures for 10 days. Single colonies that acquired the Ps^+^ phenotype (photosynthetic competence) were isolated, and reversion mutations were identified by sequencing the entire *pet*ABC operon.

##### Isolation and Purification of Cytochrome bc_1_ Complexes

Chromatophores from *R. capsulatus* cells grown under semiaerobic conditions were obtained using the procedure described previously (38). Cytochrome *bc*_1_ complexes were isolated from detergent-solubilized chromatophores using ion exchange chromatography (DEAE-BioGel A) as described (38).

##### Steady-state Kinetics Measurements

Steady-state enzymatic activity of isolated cytochrome *bc*_1_ complexes was determined spectroscopically by the 2,3-dimethoxy-5-methyl-6-decyl-1,4-benzohydroquinone-dependent reduction of cytochrome *c* (bovine heart cytochrome *c* from Sigma-Aldrich) as described previously (36). All enzymatic assays were performed in 50 mm Tris buffer (pH 8) containing 0.01% *n*-dodecyl β-d-maltoside and 100 mm NaCl. The final concentrations of substrates were as described in Table 1 and the legend to Fig. 8. Concentrations of cytochrome *bc*_1_ were in the range of 10–100 nm depending on activity of the mutant. Turnover rates were calculated from the initial linear parts of the curves. The level of superoxide production was expressed as percent difference of enzymatic activity in the presence and absence of 100 units/ml CuZn-SOD (20, 25, 26). Student's *t* test for independent samples was used for statistical analysis. Only *p* values lower than 0.05 were considered statistically significant.

Superoxide radical production was also measured via hydrogen peroxide formation using the Amplex Red horseradish peroxidase method (Amplex Red Hydrogen Peroxide/Peroxidase Assay Kit, Life Technologies). Assays were performed in 50 mm Tris buffer (pH 8) containing 0.01% *n*-dodecyl β-d-maltoside and 100 mm NaCl. The final concentrations of substrates were as described in the legend to Fig. 9. The reaction mixture also contained 50 μm Amplex Red reagent, 0.1 unit/ml HRP, and 300 units/ml CuZn-SOD. CuZn-SOD was added in excess to convert superoxide into hydrogen peroxide, which in the presence of horseradish peroxidase reacts with Amplex Red reagent to produce the red fluorescent oxidation product resorufin. Resorufin has a broad absorption peak (between 500 and 590 nm) with maximum at 571 nm. The increase in resorufin absorbance was followed at the isosbestic point of cytochrome *c* at 540 nm. The levels of H_2_O_2_ were determined from the absorbance at 540 nm measured at the time point when all substrates were used up (after reduction of 10 μm cytochrome *c*).

##### Light-induced Electron Transfer Measurements

Double wavelength time-resolved spectrophotometry (39) was used to obtain the transient kinetics of heme *b* reduction at 560–570 nm. All measurements were performed using bacterial chromatophores suspended in MOPS buffer (pH 7) containing 1 mm EDTA and 100 mm KCl following the procedure described previously (39, 40). The samples were poised at an ambient potential of 100 mV in the presence of 3.5 μm valinomycin and redox mediators as described (39). Rates of flash-induced heme *b* reduction were determined by fitting transient kinetics to a single exponential equation.

##### [2Fe-2S] Cluster Relaxation Measured by Pulse EPR

The temperature dependence of phase relaxation rate of [2Fe-2S] cluster was measured in isolated complexes using pulse EPR spectroscopy. The measurements were carried out on a Bruker Elexsys-E580 spectrometer at Q-band (33.5 GHz). The electron spin echo decay of each sample was recorded in temperature range from 12 to 24 K in the same manner as described previously (41). The relaxation rates were determined from fitting a stretched exponential function to the measured electron spin echo curves. The samples were prepared in 50 mm Bicine buffer (pH 8) and 100 mm NaCl under reducing conditions (1 mm sodium ascorbate) in the presence of 20% glycerol as described previously (41). The measured relaxation rates concern intracomplex interactions and are highly reproducible, falling within a standard error typical of fitting procedure (1–2%), irrespective of protein isolation.

##### The EPR Potentiometric Titration of [2Fe-2S] Cluster

Potentiometric titrations of [2Fe-2S] cluster in chromatophore membranes were conducted as described (40, 42). Measurements were performed in 50 mm Bicine buffer (pH 8) containing 100 mm KCl, 20% glycerol, and mediators 2,3,5,6-tetramethyl-1,4-phenylenediamine, 1,2-naphthoquinone-4-sulfonate, 1,2-naphthoquinone, 2,3,5,6-tetrachlorohyroquinone, *N*-ethyldibenzopyrazine ethyl sulfate salt, and *N*-methylphenazonium methyl sulfate, each at a concentration of 100 μm. All CW EPR spectra were recorded at 20 K using the following parameters: power, 1.9 mW; frequency, 9.39 GHz; modulation amplitude, 10 G. To obtain the value of midpoint redox potential at pH 8.0 (*E_m_*_8_), the amplitude of CW EPR spectra of the reduced [2Fe-2S] cluster was plotted as a function of ambient redox potential (*E_h_*) and fitted with the Nernst equation assuming a one-electron couple.

## Results

### 

#### 

##### Spectral and Kinetic Properties of G167P in Cytochrome b

**FIGURE 2.**
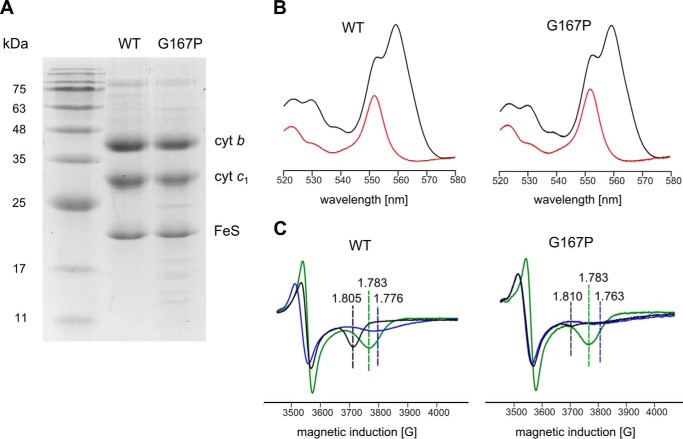
**Spectral properties and subunit composition of WT and G167P mutant.** *A*, SDS-PAGE analysis of isolated complexes. *B*, optical difference spectra of purified cytochrome (*cyt*) *bc*_1_ complexes. *Black* and *red lines* correspond to dithionite minus ferricyanide and ascorbate minus ferricyanide spectra, respectively. *C*, X-band CW EPR spectra of the [2Fe-2S] cluster measured in chromatophores suspended in 50 mm MOPS (pH 7) and 100 mm KCl. Samples were reduced with ascorbate in the absence of any inhibitor (*black* traces) and in the presence of myxothiazol (*blue* traces) or stigmatellin (*green* traces). *Dotted lines* indicate the position of the g_x_ transition. *G*, gauss.

As shown in Fig. 1*C*, Gly-167 in *R. capsulatus* cytochrome *b* is located at the end of helix *cd1* close to the twist loop that separates this helix from helix *cd2*. This region is at the entry of the Q_o_ quinol binding pocket and comes in close contact with ISP-HD when it approaches cytochrome *b*. In mitochondrial complex III, the corresponding position is occupied by Ser (Ser-151). Introducing Pro at this position (G167P) results in expression of cytochrome *bc*_1_ in *R. capsulatus* cells that contains all catalytic subunits (cytochrome *b*, cytochrome *c*_1_, and ISP) as visualized on SDS gels (Fig. 2*A*).

**FIGURE 3.**
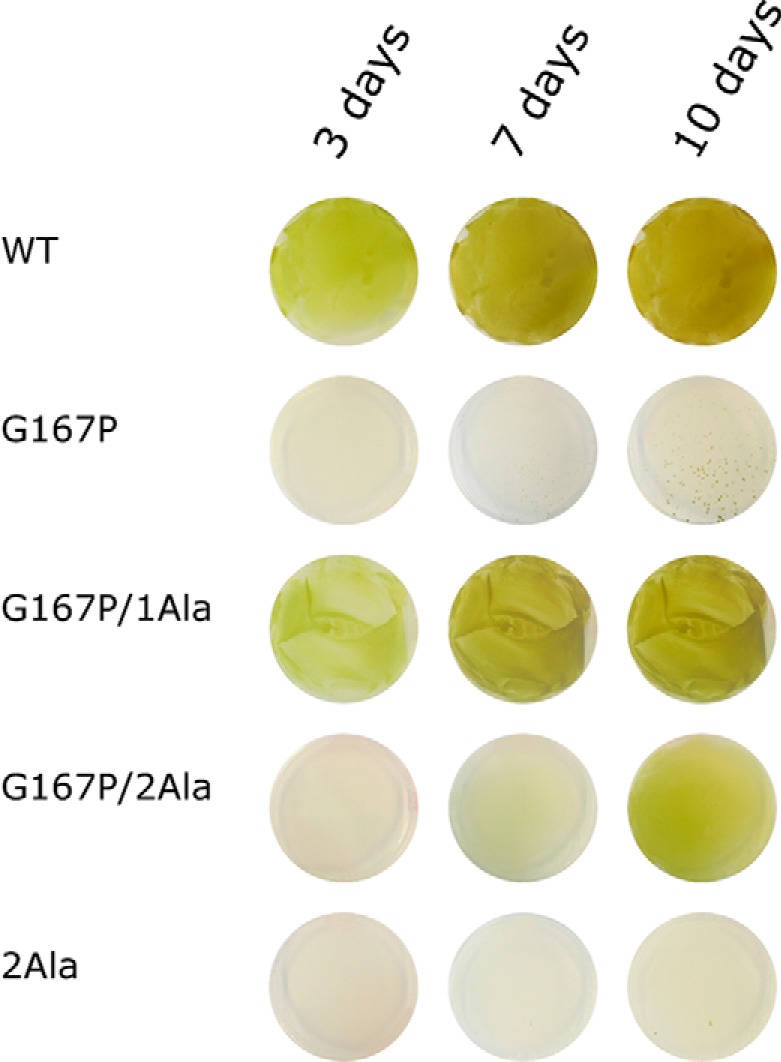
**Photosynthetic growth of various *R. capsulatus* strains.** Single *green dots* visible on the G167P plate after 10 days represent revertants (Ps^+^). Panels show growth on plates observed after 3, 7, and 10 days.

**FIGURE 4.**
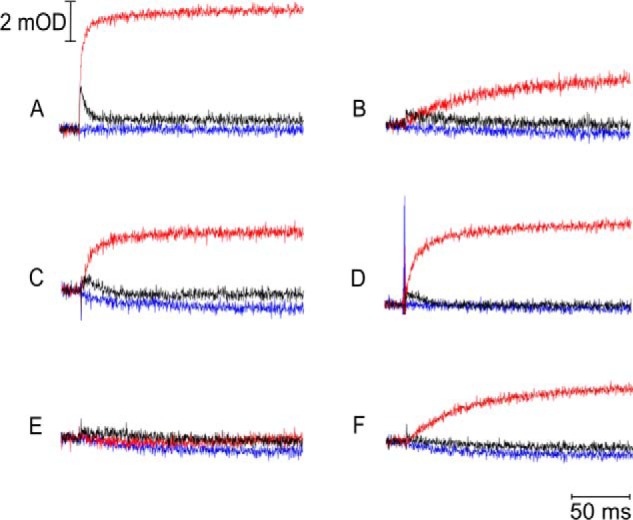
**Flash-activated heme *b* reduction.** The traces were recorded for WT (*A*), G167P (*B*), 1Ala (*C*), G167P/1Ala (*D*), 2Ala (*E*), and G167P/2Ala (*F*) at pH 7 and an ambient potential of 100 mV. Kinetic transients at 560–570 nm were recorded without inhibitors (*black lines*) and in the presence of antimycin or myxothiazol (*red* or *blue lines*, respectively). *mOD*, milli-optical density units.

However, G167P mutant is not functional *in vivo* as indicated by the incapability of cells to sustain cytochrome *bc*_1_-dependent photosynthetic growth (Fig. 3 and Table 1). This correlates with low enzymatic activity of the mutant (turnover rate of 14.7 *versus* 140 s^−1^ in WT) and severe impediments in the operation of the Q_o_ site identified by light-induced kinetic measurements. As shown in Fig. 4 (*A* and *B*, *red* trace), the Q_o_ site-mediated reduction of hemes *b* in the presence of antimycin is greatly inhibited in G167P (Table 1). In the absence of inhibitors, the reduction of hemes *b* through the Q_o_ site and reoxidation through the Q_i_ site takes place (Fig. 4, *A* and *B*, *black* trace) but because of the much slower rate of heme *b* reduction is not kinetically resolved as in WT and only visualized as the difference between the *black* and *red* traces in Fig. 4*B*. Overall, the enzymatic assays and light-induced measurements indicate severe, but not complete, inhibition of the Q_o_ site in G167P.

**TABLE 1:** **Selected properties of *R. capsulatus* mutants**

Strains	Phenotype*^a^*	Enzymatic activity*^b^*	Flash-induced heme *b* reduction*^c^*	*E_m_*_8_ of Rieske cluster	Reversions
		*s*^−1^	*s*^−1^	*mV*	
WT	Ps^+^	140 ± 5	818	308	NA*^d^*
G167P	Ps^−^	14.7 ± 0.2	16.6	290	G167Q, G167S
G167P/1Ala	Ps^+^	34 ± 2	131	293	NA
G167P/2Ala	Ps^slow^	21 ± 1	22.6	300	NA
1Ala	Ps^+^	144 ± 7	101	334	NA
2Ala	Ps^−^	4.2 ± 0.2	ND*^e^*	390	ND
G167Q	Ps^+^	70 ± 4	472	ND	NA
G167S	Ps^+^	105 ± 5	805	ND	NA

*^a^* Ps^+^ and Ps^−^ indicate photosynthetic competence and incompetence, respectively.

*^b^* Enzymatic activity rates are expressed as μmol of cytochrome *c* reduced/μmol of cytochrome *bc*_1_/s. Conditions were 50 mm Tris-HCl (pH 8), 100 mm NaCl, 20 μm quinol, and 20 μm oxidized cytochrome *c* from bovine heart. Errors represent S.D. of the mean of at least four measurements.

*^c^* Flash-induced heme *b* reduction rates for pH 7 and ambient potential of 100 mV.

*^d^* NA, not applicable.

*^e^* ND, not determined.

The optical spectra of hemes *b* and *c* in the G167P mutant do not differ from the spectra of native complex (Fig. 2*B*). They exhibit an ascorbate-reducible peak at 553 nm (reflecting high potential heme *c*_1_) and dithionite-reducible peaks at 553 and 560 nm (the latter one reflecting the low potential hemes *b*_L_ and *b*_H_). Conversely, the CW EPR spectra of the Rieske cluster in G167P clearly differed from that of the cluster in WT cytochrome *bc*_1_ (Fig. 2*C*): the g_x_ transition in the mutant is much broadened and of a smaller amplitude as compared with the sharp and characteristic g_x_ = 1.805 present in WT. In addition, the g_x_ in G167P appears to be composed of more than one component (see also the spectrum of G167P in Fig. 7). This change in the shape of the spectrum provides the first indication that the interaction between the Rieske cluster and quinone in the Q_o_ site is altered due to changes in the occupancy of the site with quinone and/or changes in the position of ISP-HD caused by structural constraints affecting the motion of this domain. Those types of changes would provide an explanation for the enzymatic and kinetic impediments observed in G167P. The g_x_ transition remains sensitive to the addition of Q_o_ site-specific inhibitors stigmatellin and myxothiazol (Fig. 2*C*). The shape of g_x_ in the presence of these inhibitors in G167P is similar to that of native complex: in both cases, stigmatellin induces sharp g_x_ at 1.783, whereas myxothiazol largely broadens this transition, shifting it to a value of g_x_ = 1.763.

##### Phase Relaxation of Rieske Cluster in G167P

**FIGURE 5.**
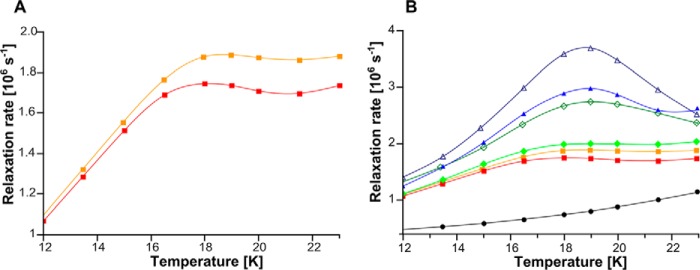
**Temperature dependence of phase relaxation rate of [2Fe-2S] cluster in native and mutated cytochrome *bc*_1_.** *A*, G167P (*red closed squares*) and WT (*orange closed squares*). *B*, 2Ala (*blue open triangles*), G167P/2Ala (*blue closed triangles*), 1Ala (*dark green open diamonds*), G167P/1Ala (*green closed diamonds*), G167P (*red closed squares*), and WT (*orange closed squares*) (last two sets of data are replotted from *A*). Control sample (*black closed circles*) is WT in which there are no dipolar interactions between heme *b*_L_ and [2Fe-2S] cluster. All samples in *A* and *B* were reduced with ascorbate except for the control of WT that was reduced with dithionite.

**FIGURE 6.**
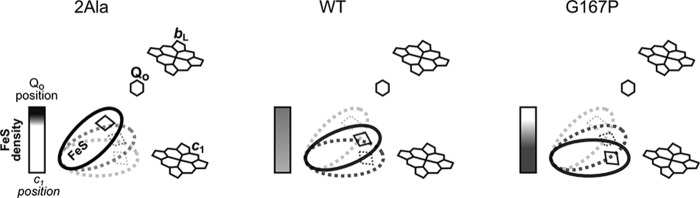
**Scheme illustrating difference in equilibrium distribution of ISP-HD position in 2Ala and G167P in comparison with WT.** In 2Ala mutant, ISP-HD stays captured at the Q_o_ site for seconds, and its average position is shifted toward the Q_o_ site in relation to WT. In the native cytochrome *bc*_1_, ISP-HD moves freely between Q_o_ and *c*_1_ positions. G167P has an effect opposite to 2Ala: the average position of ISP-HD is more remote from the Q_o_ site than in WT. *Horizontal rectangles* depict the density of [2Fe-2S] cluster at the Q_o_ site: the *darker* and *lighter shades* denote higher and lower occupancy of the [2Fe-2S] cluster, respectively.

To get further insights into the molecular effects of G167P, we performed analysis of the temperature dependence of the phase relaxation rate of the Rieske cluster. Our previous studies showed that changes in phase relaxation rate reflect changes in the position of ISP-HD (41). More specifically, at the macroscopic level of the protein solution, they reflect changes in the average equilibrium position of ISP-HD with respect to other subunits of the complex. This approach benefits from the observation that oxidized heme *b*_L_ enhances the relaxation in a distance-dependent manner: the closer the [2Fe-2S] cluster to heme *b*_L_, the stronger the enhancement. This means that the movement of ISP-HD out of the Q_o_ site resulting in an increase of distance between heme *b*_L_ and Rieske cluster weakens the enhancement (41). A comparison of the temperature dependence profiles of the phase relaxation rate of the Rieske cluster shown in Fig. 5*A* reveals that the enhancement in G167P is weaker compared with WT. This indicates that in G167P the average equilibrium position of ISP-HD is set at a larger distance from heme *b*_L_ (and the Q_o_ site) than in WT (Fig. 6, *right versus middle*). In other words, G167P causes a shift in the equilibrium position of ISP-HD toward positions more remote from the Q_o_ site.

##### Reversions

When incubated under photosynthetic conditions, a photosynthetically inactive strain carrying G167P reverts spontaneously to photosynthetically competent cells (see Fig. 3). The DNA analysis of the revertant cells revealed that they are same site revertants and replace Pro at 167 with Ser or Gln (of eight clones analyzed, seven introduced Ser (generating mutant G167S) and one introduced Gln (generating mutant G167Q)). The enzymatic assays and kinetic data indicated that both G167S and G167Q regain much of their electron transfer activity compared with the original G167P. However, compared with WT, G167Q still exhibits about 2 times slower enzymatic turnover and rate of flash-induced heme *b* reduction, whereas G167S in both measurements approaches the WT level (Table 1). It is of note that position 167 is naturally occupied by Ser in several species including humans (corresponding position is Ser-151). High electron transfer activity of G167S is consistent with this observation.

##### Effects of Combination of G167P in Cytochrome b with 1Ala and 2Ala Insertions in the Neck Region of ISP Subunit

It was previously recognized that alanine insertion (1Ala or 2Ala) in the neck region connecting the ISP-HD with its hydrophobic anchor introduces steric constrains to the motion of ISP-HD (10). As a result, ISP-HD stays captured at the Q_o_ site for milliseconds in 1Ala mutant or seconds in 2Ala mutant, which is a much longer time than in WT (microseconds or less) (10). Therefore, in solution containing 1Ala or 2Ala mutants, there is an increase in the population of complexes having ISP-HD at the Q_o_ site (25, 41). In this context, a direction of the shift in the average position of ISP-HD caused by G167P can be considered opposite to the one caused by 1Ala or 2Ala mutants (Fig. 6). This prompted us to analyze the effect of a combination of G167P and alanine insertions in two double mutants, G167P/1Ala and G167P/2Ala. As shown in Fig. 3 and Table 1, both mutants, unlike G167P, can grow under photosynthetic conditions. Clearly, either 1Ala or 2Ala can suppress effects caused by G167P. However, the growth of G167P/1Ala is more vigorous than that of G167P/2Ala, indicating that the former combination of mutations yields cytochrome *bc*_1_ that operates more efficiently. This difference finds its roots in the different phenotypic properties of the original Ala insertions (10): 1Ala is photosynthetically active, whereas 2Ala is not (Table 1). Thus, although in the case of G167P/1Ala an addition of the Ps^+^ mutation (1Ala) to the Ps^−^ mutation (G167P) rendered the double mutant Ps^+^, in the case of G167P/2Ala, a weak Ps^+^ phenotype was achieved by combining the two mutations that originally were Ps^−^ (Fig. 3 and Table 1).

The enzymatic activities and measured rates of light-induced electron transfer in the double mutants seem consistent with their phenotypic properties (Fig. 4 and Table 1). G167P/2Ala shows a slight increase in the turnover rate and rate of light-induced heme *b* reduction as compared with G167P. However, changes in G167P/2Ala are more dramatic in relation to the 2Ala mutant, which has a very low enzymatic activity (turnover of around 4 s^−1^) and no signs of millisecond heme *b* reduction under the conditions described in Fig. 4 (G167P/2Ala displays a 5 times higher turnover rate and a clear millisecond reduction of heme *b*). G167P/1Ala shows a further increase in both the enzymatic activities and heme *b* reduction compared with G167P/2Ala (Table 1). However, the extent of the increase is clearly larger for heme *b* reduction than for enzymatic activity. The same trend is also observed when G167P/1Ala is compared with G167P: the rate of flash-induced heme *b* reduction increases about an order of magnitude, whereas the enzymatic activity increases only 2 times (Table 1). In fact, the rate of heme *b* reduction in G167P/1Ala reaches the level observed in 1Ala; however, the enzymatic activity of 1Ala is about 4 times higher compared with G167P/1Ala. This makes G167P/1Ala an interesting example of a combination of mutations exerting more severe inhibitory effects under the conditions of multiple turnover than under the conditions of flash-induced electron transfer.

**FIGURE 7.**
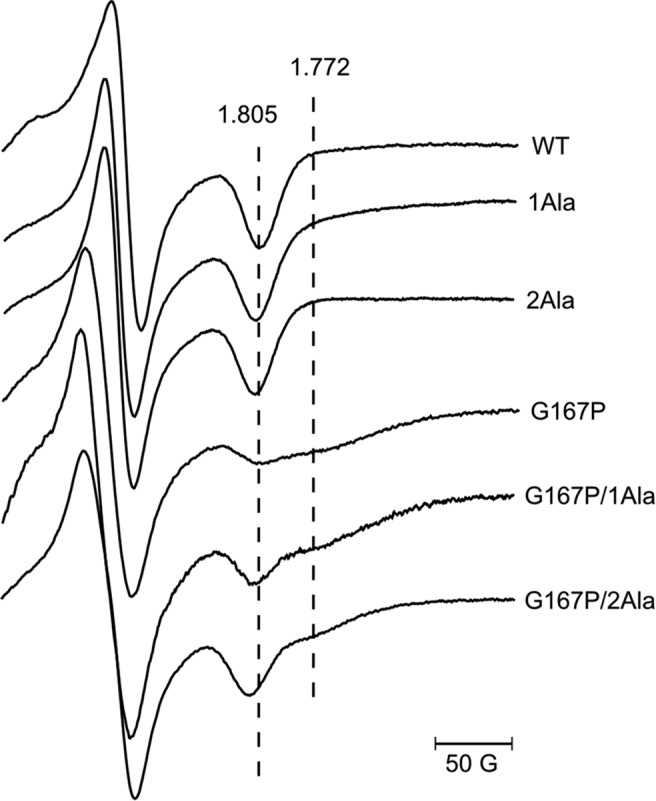
**X-band CW EPR spectra of [2Fe-2S] cluster of WT and various mutants.** All EPR spectra were measured in chromatophores suspended in 50 mm MOPS (pH 7) and 100 mm KCl and reduced with ascorbate. *Dotted lines* indicate the position of [2Fe-2S] g_x_ transitions.

As seen in the EPR spectra of Fig. 7, the heterogeneity of g_x_ transition originally observed in G167P is more pronounced in G167P/1Ala and G167P/2Ala. The g_x_ in EPR spectra of both double mutants allows for the distinction of two transitions (1.805 and 1.772). If g_x_ = 1.805 is taken into account, one can see an increase in the amplitude in the following order: G167P, G167P/1Ala, G167P/2Ala, WT. As the characteristic shape of g_x_ = 1.805 in WT is usually assigned as reflecting interaction of reduced [2Fe-2S] cluster with quinone bound in the Q_o_ site (43, 44), the increase in the amplitude of g_x_ = 1.805 in double mutants in comparison with G167P suggests that in those mutants the population of ISP-HD occupying the Q_o_ position is larger. This seems to follow the expectations based on the opposing effects of 1Ala or 2Ala and G167P. However, it is important to emphasize that the shape of the EPR spectrum in chromatophores frozen without glycerol may not necessarily reflect the distribution of positions of ISP-HD as best evidenced in 1Ala and 2Ala mutants, which both have EPR spectra similar in shape to WT but exert large effects on distribution of ISP-HD positions (Figs. 5*B* and 7) (25, 41, 45). Conversely, the increase in g_x_ in double mutants points toward the possibility that the change in the shape of EPR spectrum of G167P alone (Fig. 7) is caused by the remoteness of ISP-HD from the Q_o_ site rather than the absence of quinone bound at the Q_o_ site (provided that 2Ala or 1Ala does not change the affinity of Q_o_ site for its substrate in G167P).

To get insights into the effects of G167P/1Ala and G167P/2Ala on the distribution of positions of ISP-HD, we compared the temperature dependence profiles of the phase relaxation rate of the Rieske cluster in those mutants, taking the profiles of 2Ala, 1Ala, and WT as a reference (Fig. 5*B*). The strongest enhancement observed in the 2Ala mutant reflects almost the entire population of ISP-HD at the Q_o_ site. The enhancement in G167P/2Ala is weaker, indicating a decrease in the population of ISP-HD at the Q_o_ site in this mutant. However, it stays above the level of enhancement seen in the 1Ala mutant, indicating that the population of ISP-HD at the Q_o_ site in G167P/2Ala is larger than in 1Ala. The enhancement in G167P/1Ala is weaker than in 1Ala, indicating a further decrease in the population of ISP-HD at the Q_o_ site. This population is still larger than in WT (enhancement in G167P/1Ala is larger).

Overall, the decrease in the strength of the enhancement indicates a decrease in the population of ISP-HD at the Q_o_ site and a shift in equilibrium position toward positions more remote from the Q_o_ site. It appears as if on one hand 1Ala and 2Ala diminished the “pushing” of the ISP-HD out of the Q_o_ site caused by G167P, and on the other hand, G167P diminished the pushing of the ISP-HD toward the Q_o_ site caused by 1Ala and 2Ala. As a result, the average ISP-HD position in G167P/1Ala or G167P/2Ala became closer to the Q_o_ site than in WT (and G167P) but did not reach proximity to the Q_o_ site achieved in 1Ala or 2Ala, respectively. Considering all these effects, one can rank the mutations in the following order (with the first having the largest population of ISP-HD at the Q_o_ site): 2Ala > G167P/2Ala > 1Ala > G167P/1Ala > WT > G167P.

Table 1 shows that values of midpoint redox potential (*E_m_*) of the Rieske cluster in the mutants G167P, G167P/1Ala, and G167P/2Ala are similar to that of WT. This differentiates these mutants from the 1Ala and 2Ala mutants, which were previously shown to display an increase in *E_m_* of Rieske cluster in respect to WT (in particular, 2Ala displays an *E_m_* value about 100 mV higher than WT) (10). Given that the Ala mutants generally increase the population of ISP-HD at the Q_o_ site, their effect on *E_m_* is consistent with the notion that the Rieske cluster in/close to the Q_o_ site has a higher *E_m_* if compared with its *E_m_* at other positions (46) (an increase in *E_m_* is also observed in the presence of some inhibitors such as stigmatellin (47) that fix the ISP-HD at the Q_o_ site). In this context, the lack of an increase in *E_m_*_8_ of G167P, G167P/1Ala, and G167P/2Ala can be considered as additional support to the notion that a population of ISP-HD at the Q_o_ site is generally decreased in all G167P mutants (*i.e.* even when Ala insertions are present). We note, however, that the origin of the changes in *E_m_* of the Rieske cluster is a complex issue as *E_m_* not only depends on the position of ISP-HD but on several other factors including occupancy of the Q_o_ site with substrate/inhibitor (45, 48). Thus, a change in a value of *E_m_* is not a simple translation to changes in ISP-HD position. A good example is provided by G167P/2Ala, which has a larger population of ISP-HD at the Q_o_ site than 1Ala (Fig. 5*B*) but *E_m_*_8_ of Rieske cluster is not elevated (Table 1).

##### Effect of G167P on Generation of Superoxide at the Q_o_ Site of Cytochrome bc_1_

It was previously recognized that superoxide is generated at the Q_o_ site in reactions involving a back electron transfer from heme *b*_L_ to quinone (24–26). This leads to the formation of semiquinone, which has the highest probability of reacting with oxygen when ISP-HD occupies a position remote from the Q_o_ site (25, 26). It follows that mutations shifting the average equilibrium position of ISP-HD out of the Q_o_ site should in principle enhance superoxide production by cytochrome *bc*_1_. G167P seemed a good candidate to test this assumption.

**FIGURE 8.**
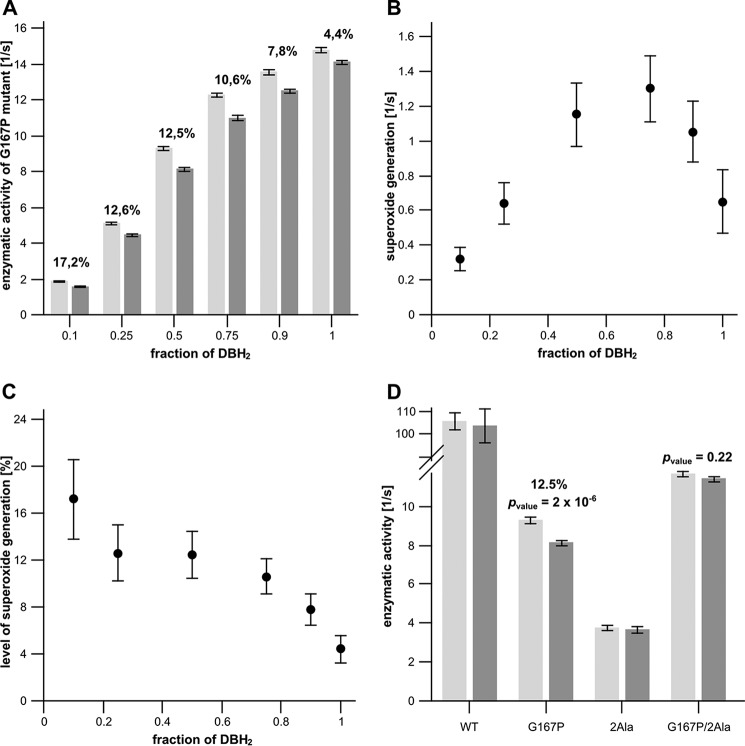
*A*, enzymatic activities of uninhibited G167P mutant under varying redox states of the quinone pool (quinol/quinone pool ratio) in the absence (*light gray bars*) and presence (*dark gray bars*) of SOD. Conditions were 50 mm Tris-HCl (pH 8), 100 mm NaCl, and 20 μm oxidized cytochrome *c*. The total concentration of the quinone pool was 20 μm. The amount of superoxide production is shown as a percentage *above* the *bars. Error bars* represent S.D. of the mean of 12 measurements. *B*, rates of superoxide generation by G167P calculated from the data in *A. C*, percentage of SOD-sensitive cytochrome *c* reduction in relation to cytochrome *c* reduction measured in the absence of SOD (based on the data of *A*). *D*, enzymatic activities of WT and mutants (G167P, 2Ala, and G167P/2Ala) measured in the absence (*light gray bars*) and presence (*dark gray bars*) of SOD. Conditions were 50 mm Tris-HCl (pH 8), 100 mm NaCl, 10 μm quinol, 10 μm quinone, and 20 μm oxidized cytochrome *c*. Only a statistically significant amount of superoxide production is shown as a percentage *above* the *bars. Error bars* represent S.D. of the mean of 12 measurements. *DBH_2_*, reduced form of 2,3-dimethoxy-5-methyl-6-decyl-1,4-benzoquinone.

Typically, production of superoxide in native cytochrome *bc*_1_ is observed when the inhibitor antimycin blocks the reoxidation of heme *b*_H_ (and *b*_L_) via the Q_i_ site (20, 26, 49). The measurements are performed in detergent solution with isolated enzyme exposed to an excess of substrate, cytochrome *c* and quinol. Our initial measurements indicated that under those types of conditions the G167P mutant generates even larger amounts of superoxide than the native cytochrome *bc*_1_ (30 *versus* 17% for G167P and WT, respectively). This prompted us to verify whether this mutant can generate superoxide in the absence of any inhibitor. However, the measurements performed with an excess of quinol (typical conditions of enzymatic assays) revealed only a little superoxide generated by G167P (less than 5%). We thus tested a variety of conditions where both quinol and quinone were present in the reaction mixture in various proportions. The results are shown in Fig. 8. The amount of superoxide generation is described “quantitatively” as a number of μmol of O_2_^˙̄^ produced by 1 μmol of the enzyme per second (Fig. 8*B*) or “relatively” as a percentage of superoxide generated per single turnover (Fig. 8*C*). Quantitatively, the superoxide generation shows a bell shape with the maximum at a quinol to quinone pool ratio of ∼70 to 30%. Changes of this proportion in any direction lead to a decrease of the total amount of superoxide generated per second. Relatively, a linear increase of superoxide generation is observed upon an increase of quinone and decrease of quinol. The bell shape of O_2_^˙̄^ generation (Fig. 8*B*) is a consequence of a balance between two opposite effects associated with oxidation of the Q pool: O_2_^˙̄^ per single turnover increases with increasing concentration of quinone, while at the same time, the total enzymatic turnover rate decreases (Fig. 8*A*).

Under the conditions of maximum superoxide production per second observed for G167P, WT and mutants 2Ala and G167P/2Ala did not generate superoxide within experimental uncertainty (±S.E.) (Fig. 8*D*). The difference between mean values of enzymatic activities with and without SOD is shown for G167P (*p* value = 2 × 10^−6^, statistically significant result) and G167P/2Ala (*p* value = 0.22, not statistically significant result). The results for WT and 2Ala are consistent with previous observations described (26).

**FIGURE 9.**
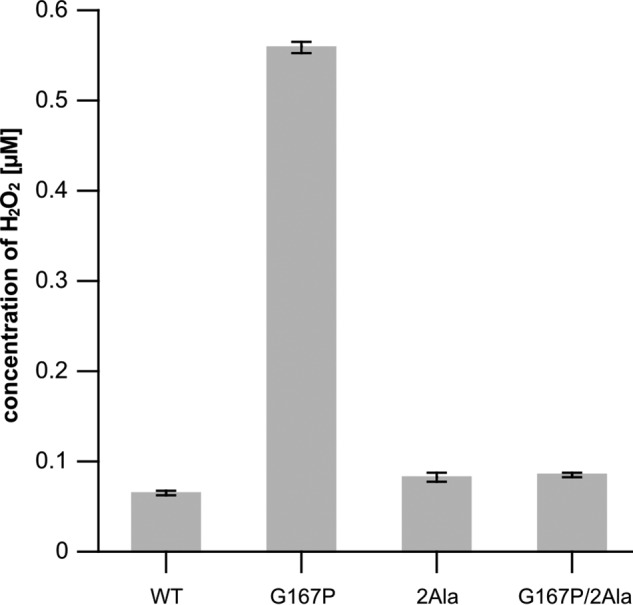
**Concentration of H_2_O_2_ accumulated during the course of the reaction catalyzed by WT cytochrome *bc*_1_ and mutants (G167P, 2Ala, and G167P/2Ala) in the presence of SOD measured using the Amplex Red horseradish peroxidase method.** Conditions were 50 mm Tris-HCl (pH 8), 100 mm NaCl, 10 μm quinol, 10 μm quinone, and 10 μm oxidized cytochrome *c* from bovine heart. *Error bars* represent S.D. of the mean of four measurements.

To confirm enhanced production of superoxide in G167P mutant, we performed additional analysis using the Amplex Red horseradish peroxidase method (Fig. 9). We also observed that uninhibited G167P mutant produces a high level of superoxide, which was reflected in 10 times higher concentration of H_2_O_2_ measured for this mutant with respect to WT, 2Ala, or G167P/2Ala. We note that the background level of H_2_O_2_ observed for WT, 2Ala, and G167P/2Ala is consistent with the results reported for uninhibited complex III (24).

## Discussion

Replacement of Gly-167 in cytochrome *b* with Pro is expected to result in structural distortions in the region encompassing helix *cd1* and helix *cd2*, for example in a change in relative position of these two helices. As this region forms a part of the interaction site of cytochrome *b* with ISP-HD (7, 50, 51), such distortions may influence the process of binding/release of ISP-HD upon its interaction with cytochrome *b*. This will impact the motion of ISP-HD, which will manifest itself in the change in the equilibrium position of ISP-HD. Indeed, the relaxation enhancement measurements revealed that the average equilibrium position of ISP-HD in G167P is at a larger distance from heme *b*_L_ if compared with WT (Fig. 5*A*). This provides the first (to our knowledge) example of a mutation in which the shift of position of ISP-HD out of the Q_o_ site was documented spectroscopically. So far, the documented and well characterized cases concerned mutations that shift the average position of ISP-HD in the opposite direction; *i.e.* the ISP-HD is arrested at the Q_o_ site, which makes the average equilibrium position of ISP-HD at a closer distance from heme *b*_L_ (if compared with WT). The most prominent mutations that act in this way are insertions in the neck region of the ISP subunit (in particular 1Ala and 2Ala mutants, which arrest the ISP-HD for milliseconds or seconds, respectively) (10, 25, 41).

The effects of G167P on the distribution of ISP-HD caused a large decrease in the rates of electron transfer and enzymatic activity (Table 1), which rendered cytochrome *bc*_1_ non-functional *in vivo* (the enzyme was not able to support growth of the cells under photosynthetic conditions) (Fig. 3). Interestingly, a simultaneous presence of the oppositely acting 1Ala or 2Ala mutant alleviates the effects of G167P to the point that *in vivo* functionality of cytochrome *bc*_1_ is restored. However, as the original 1Ala and 2Ala mutants affect the motion of ISP-HD to various degrees (10), the overall effects of a combination of G167P with 1Ala or 2Ala (G167P/1Ala or G167P/2Ala, respectively) differed. G167P/1Ala displayed generally higher activities and better growth than G167P/2Ala, which in terms of measured *in vitro* activities was only slightly better than G167P and consequently displayed rather weak photosynthetic growth (compared with G167P/1Ala or WT) (Table 1 and Fig. 3).

EPR analysis revealed that the average position of ISP-HD in G167P/2Ala and G167P/1Ala sets at a longer distance from heme *b*_L_ than in the respective 2Ala and 1Ala mutants but still shorter than distances in G167P and WT (Fig. 5*B*). A new average position of ISP-HD for each double mutant (falling between the two average positions of ISP-HD in single mutants) indicates that the effects of two oppositely acting mutations (G167P *versus* 1Ala or 2Ala) add. The new positions extend the array of ISP-HD positions available for functional/structural studies.

Based on the results obtained with G167P and with double mutants G167P/2Ala and G167P/1Ala, we anticipate that there are other mutations in cytochrome *b* likely to exert an effect similar to that of G167P. The prominent candidates are mutations in the *ef* loop region of cytochrome *b* (such as L286F) identified as suppressors of 1Ala mutation (52). The suppression effect would be analogical to the effect of combination of G167P with 1Ala described here. Although the *ef* loop is in a different region of cytochrome *b* than helix *cd1*/*cd2*, it also forms a part of the interaction site of cytochrome *b* with ISP-HD. Furthermore, the *ef* loop was proposed to form a barrier that ISP-HD needs to cross to move out of the Q_o_ site upon its large scale movement toward cytochrome *c*_1_ and to move back to the site (52). The suppression mutation L286F was proposed to diminish the barrier, facilitating the movement of ISP-HD in the presence of 1Ala (52). Conceivably, this would shift the average position of ISP-HD toward positions more remote from the Q_o_ site as in G167P. However, the influence of L286F on motion of ISP-HD is not as large as in G167P as indicated by less severe kinetic impediments of L286F in comparison with G167P and by the Ps^+^ phenotype of the L286F mutant (as opposed to Ps^−^ G167P) (52). Another indication that the effects of G167P might be more profound than those of L286F comes from the observation that only G167P was able to suppress the effects of 2Ala.

G167P offered an important set of new conditions for investigating the mechanism of superoxide production by cytochrome *bc*_1_. According to a recently proposed mechanism, the back electron transfer from reduced heme *b*_L_ to quinone (24, 25) results in formation of semiquinone at the Q_o_ site that has the best chance to react with oxygen when the ISP-HD occupies positions remote from the Q_o_ site at the time of semiquinone formation. The kinetic constraint associated with the position of ISP-HD was originally deduced from the observation that mutants arresting ISP-HD at the Q_o_ site (2Ala and 1Ala) suppress ROS production by cytochrome *bc*_1_ (25, 26). This model predicts that mutations that act in the opposite direction, *i.e.* shift the equilibrium position of ISP-HD toward a position more remote from the Q_o_ site, should increase the level of ROS production. The results obtained with G167P are consistent with this prediction. Antimycin-inhibited G167P showed an increased level of superoxide production in comparison with antimycin-inhibited native enzyme. Furthermore, G167P generated superoxide even without any inhibitor present when the reaction mixture contained quinone in addition to quinol in the reaction mixture (Figs. 8 and 9). This was in contrast to the uninhibited native enzyme, 2Ala mutant, and G167P/2Ala double mutant, which did not generate detectable superoxide under any of the tested conditions (*i.e.* even with quinone present) in the measurements based on superoxide dismutase-sensitive reduction of cytochrome *c*.

The observation that it was necessary to add quinone to observe the generation of superoxide in uninhibited G167P is particularly interesting as it provides additional support for the notion that the initial reaction in the sequence leading to superoxide production is the electron transfer from heme *b*_L_ to quinone to form semiquinone in the Q_o_ site (24, 25). In this reaction, quinone acts as a substrate, explaining the negative slope of Fig. 8*C* (decrease in the level of superoxide generation with an increase in the quinol/quinone pool ratio). We note that it would be difficult to explain this slope on the basis of an alternative model assuming that the superoxide-generating semiquinone is formed upon one-electron reduction of Rieske cluster by quinol (in this case quinol, not quinone, is the substrate) (20, 21, 53, 54).

The conditions identified here under which uninhibited G167P generated superoxide are consistent with the observation reported earlier that the maximum rate of superoxide generation by complex III in mitochondrial system occurred when the Q pool was partly oxidized (24, 55). In fact, this finding was taken as initial evidence for the heme *b*_L_ to quinone reaction at the Q_o_ site (24). In those studies as in the majority of other studies, the electron flow through cytochrome *bc*_1_ must have been inhibited by antimycin to observe superoxide production. In this context, G167P shows that certain mutations in cytochrome *b* (especially those that promote ISP-HD to occupy positions remote from the Q_o_ site) may induce changes that enhance superoxide generation by cytochrome *bc*_1_ to the point that it becomes detectable in the non-inhibited enzyme. However, in our *in vitro* experiments, this appeared to strongly depend on the quinone/quinol ratio. Relating this to physiological conditions implicates that the level of generation of superoxide by certain mutants may depend on the redox state of the Q pool with a general tendency that the level will increase as the pool becomes more oxidized. It should be noted that the mutants themselves may influence the redox state of the Q pool; however, predicting a direction of this change (whether the Q pool becomes more oxidized or more reduced) is difficult. This is because such a change will be an overall effect of not just decreased enzymatic activity of the mutants (a typical effect also seen in G167P) but other factors as well (respiration rate, activity of other respiratory complexes, and membrane potential).

Remarkably, ∼70% of ubiquinol identified as the condition associated with the maximum level of superoxide production in G167P (Fig. 8*B*) closely resembles the condition of maximal superoxide production reported for antimycin-inhibited submitochondrial particles (24). This shows that modulation of superoxide production by changes in the quinol/quinone ratio can be similar in both mitochondrial and bacterial complexes irrespective of the initial cause that led to the impediment in electron transfer (mutation or inhibitor). In higher organisms, this modulation could be a part of redox signaling with the Q_o_ site acting as a sensor of the redox state of the Q pool in membrane (1, 55, 56).

S151P (G167P in *R. capsulatus*) was originally reported in a patient with exercise intolerance (35). It was restricted to muscle tissue (80% heteroplasmy) with extremely decreased complex III enzymatic activity (35). Analysis of mitochondrial particles obtained from these cells indicated a slight decrease in the amounts of cytochrome *b* and cytochrome *c*_1_ subunits (35). Conversely, the analogous mutation studied in a yeast model system resulted in a dramatic decrease in the level of ISP subunit and consequently in the enzymatic activity of the mutated complexes (34). Our results in a bacterial model system indicate that Pro at position 167 (151 in the human mitochondrial cytochrome *b*) causes a shift in the average position of ISP-HD toward positions more remote from the Q_o_ site. We propose that this effect could decrease the stability of complex III during biochemical preparations, for example by making it more susceptible to proteolytic digestion, which would in part account for the reduced amounts of various subunits observed in human tissues and the yeast model.

Interestingly, it was reported that in yeast the effect of S152P can be suppressed by a mutation, A90D (34), located in the hinge region of the ISP subunit. A90D in yeast is in the same region as Ala insertions in *R. capsulatus* (10) and thus may influence the motion of ISP-HD in analogy to the effects of 1Ala or 2Ala mutant. The observation that the effects of S152P (G167P) can be suppressed by the mutations located in the hinge region of the ISP subunit in both yeast and bacterial cytochrome *bc*_1_ comes as additional support for the notion that the original mutation influences the movement of ISP-HD, resulting in the change of its average position.

We anticipate that there might be other human disease-related mutations in cytochrome *b* at the cytochrome *b*/ISP-HD interface that cause a shift in the average position of ISP-HD in a similar direction as G167P (ISP-HD more remote from the Q_o_ site). Such mutations are likely to influence the superoxide-generating activity of complex III in a manner similar to G167P. Quite importantly, as G167P indicates here, this activity strongly depends on the redox state of the quinone pool (quinone/quinol ratio) and therefore may dynamically change in living cells.

## Author Contributions

A. B. designed research and performed optical measurements (spectra, enzymatic activities, and ROS production), photosynthetic growth experiments, analyzed and interpreted the data, and assisted in writing the paper. P. K. performed CW EPR measurements and light-induced electron transfer, analyzed and interpreted the data, and assisted in writing the paper; R. E. performed molecular biology and gel analysis and contributed to photosynthetic growth experiments. R. P. contributed to CW EPR measurements and performed statistical analysis. M. S. performed pulse EPR measurements and contributed to analysis and interpretation of the data. A. O. designed research, analyzed and interpreted the data, and wrote the paper. All authors reviewed the results and approved the final version of the manuscript.

## Footnotes

3 The abbreviations used are:

ISP: iron-sulfur protein
ISP-HD: head domain of iron-sulfur protein
WT: wild type
O_2_^˙̄^: superoxide ion
SOD: superoxide dismutase
CuZn-SOD: copper- and zinc-containing superoxide dismutase
Bicine: *N*,*N*-bis(2-hydroxyethyl)glycine
CW: continuous wave
*E_m8_*: midpoint redox potential at pH 8.0
ROS: reactive oxygen species.
